# Effect of debonding on stress indicators in cows and calves in a cow-calf contact system

**DOI:** 10.3168/jdsc.2023-0468

**Published:** 2024-01-15

**Authors:** Julie Føske Johnsen, Johanne Sørby, Sabine Ferneborg, Stine Grønmo Kischel

**Affiliations:** 1 Section of Terrestrial Animal Health and Welfare, Norwegian Veterinary Institute, 1431 Ås, Norway; 2 Department of Animal and Aquacultural Sciences, Faculty of Biosciences, Norwegian University of Life Sciences, 1432 Ås, Norway; 3 Department of Research and Development, Farm Advisory Services, TINE SA, 1432 Ås, Norway

## Abstract

Premature separation, entailing a mixture of stressful events, follows a period of cow-calf contact in dairy production. We hypothesized that timing and length of debonding (i.e., gradually adapting cow and calf to separation) may affect behavioral responses indicating stress and reinstatement of the cow-calf bond. Initially, cows had 24 h/d smart-gate access to their calves before reduction to 12 h/d, 6 h/d, and finally 0 h/d, either commencing at 4 wk after birth over 28 d: long debonding (LDB, n = 16 pairs), or at 6.5 wk, over 10 d: short debonding (SDB, n = 14 pairs). We aimed to compare the vocal response (high- and low-pitched vocalizations) and proximity (<1 m to separation barrier) of LDB versus SDB cows and calves at each reduction of access and finally at 0 h/d contact. We also aimed to assess the impact of calf supplemental milk intake on their vocal behavior. Direct observations (2 h × 2 d) were performed during the following different phases: 24 h/d access (baseline, BL24h), once cow access was reduced (separation, SEP12h), once pairs were habituated to the change (BL12h), after cow access was further reduced (SEP6h), once pairs were habituated (BL6h), and at 0 h/d contact (SEP0h). Mixed effect linear regression models indicate that overall, treatment did not affect cow behaviors nor the main indicator of separation stress, high-pitched vocalizations. Both calves and cows responded with vocal and reinstatement behavior once cows' access was reduced, with great individual variation. The LDB calves spent less time close to separation barrier and emitted more low-pitched vocalizations. Otherwise, the effect of treatment differed with phase: SDB calves emitted more high-pitched vocalizations in response to the initial reductions of cow access than LDB calves. The LDB cows (but not calves) produced more high- and low-pitched vocalizations when access was reduced to 6 h/d and 0 h/d. Calves drinking >1.5 L of supplemental milk/d emitted fewer high-pitched vocalizations. In conclusion, a longer debonding period initiated at a lower age before separation may alleviate the initial behavioral response to separation, especially for calves. The vocal response of calves increases with age at separation but is modulated by intake of supplemental milk. For cows, our results indicate that the studied debonding methods affected the behavioral response to separation only to a limited degree.

## Highlights

• Can different debonding methods alleviate stress at cow-calf separation?
• All animals, to different extents, were vocal and performed reinstatement behavior.
• Calves with a shorter debonding period showed a more pronounced acute stress reaction.
• The studied debonding methods had limited effect on the cows' stress response.
• Calves consuming supplemental milk during separation were less stressed.

## Graphical Abstract

**Figure:**
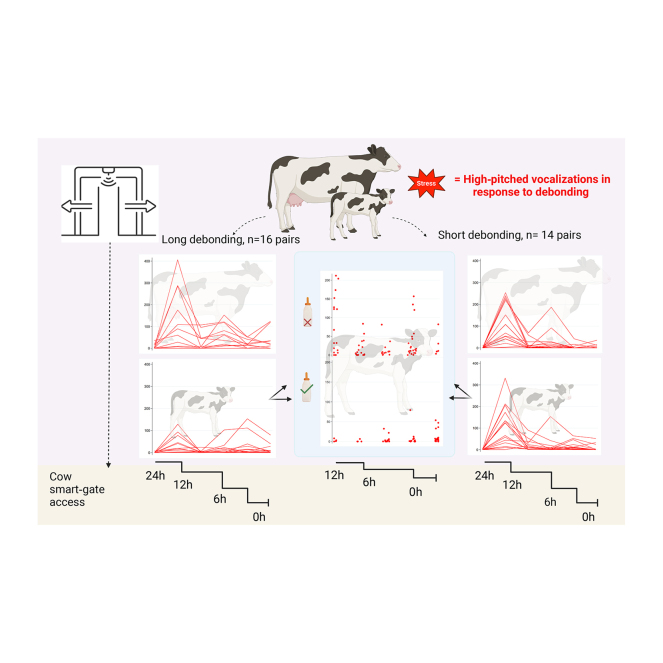
**Summary** We compared the behavioral response of cows and calves allowed either a long (28 days) or short (10 days) debonding (= 0 hours cow access). The individual, high-pitched vocalizations over treatment and smartgate access indicate great individual variation. For calves, initiation of debonding slowly and at an early age may mitigate some of the behavioral responses to separation; the vocal response is modulated by intake of supplemental milk. For cows, our results indicate that the adaptation period had a limited effect on the cows' stress response.

The latest EFSA statement on the welfare of calves strongly supports that dairy calves should be provided contact with the dam, and research on its implementation is encouraged (EFSA Panel on Animal Health and Animal Welfare et al., 2023). While various cow-calf contact (**CCC**) systems are described (Sirovnik et al., 2020), it is established that the acute behavioral responses of cows and calves to separation are more intense and prolonged when separation is delayed (e.g., Flower and Weary, 2001). Debonding refers to any process of gradually adapting cow and calf to separation. A period of debonding before (full physical) separation may aid alleviation of stress through habituation and feeding supplemental milk (Johnsen et al., 2018). In the specific example of cow-directed CCC systems, debonding may be facilitated through a gradual decrease in the cows' opportunity to visit the calf. This may encourage calf nutritional independence and habituating cow and calf to being separated. As predicted by the parent-offspring conflict hypothesis (Trivers, 1974), the interests of the mother and the offspring largely cohere when the offspring is young but diverge at weaning. Similarly, natural behaviors associated with suckling and allogrooming decrease with calf age. However, optimal length and timing of debonding is unknown. High-pitched vocalizations in cattle may signal negative emotional states and often precede cow-calf reunion (Padilla de la Torre et al., 2015; Schnaider et al., 2022) and may thus be used as an indicator of separation stress. Low-pitched vocalizations, on the other hand, are emitted when cow and calf are close to each other, and are considered a potential indicator of a positive experience (Kiley, 1972; Schnaider et al., 2022). We aimed to study short and long gradual debonding methods and compare behavioral responses of cow and calf and assess the impact of calf intake of supplemental milk on their vocal behavior.

Data were collected in a parallel group designed controlled study. Four batches of each 8 cow-calf pairs were included at birth. The Norwegian University of Life Sciences' Institutional Animal Care and Use Committee approved all procedures with animals. Each batch was semi-randomly assigned to 1 of 2 treatments: long debonding (**LDB**, n = 16 pairs) and short debonding (**SDB**, n = 14 pairs). In a cow-driven CCC system, cows moved from a cow area to visit the calves in a meeting area to which calves had access from a calf creep. Two computer-controlled smart gates facilitated the cows' entry and exit to the meeting area which initially was free 24 h/d (LDB = 4 wk, SDB = 6.5 wk) and then gradually reduced through 12 h/d access (LDB = 14 d, SDB = 5 d), 6 h/d access (LDB = 14 d, SDB = 5 d) and finally to 0 h/d (“0 access”; 7 d for both treatments, Figure 1). Calf age at the start of debonding was 4 wk for LDB pairs and 6.5 wk for SDB pairs, whereas all pairs had 0 contact from wk 8–9. Independent of smart-gate access, cows and calves were always provided fence-line contact at the separation barrier demarcating the meeting area from the cow area.

**Figure 1:**
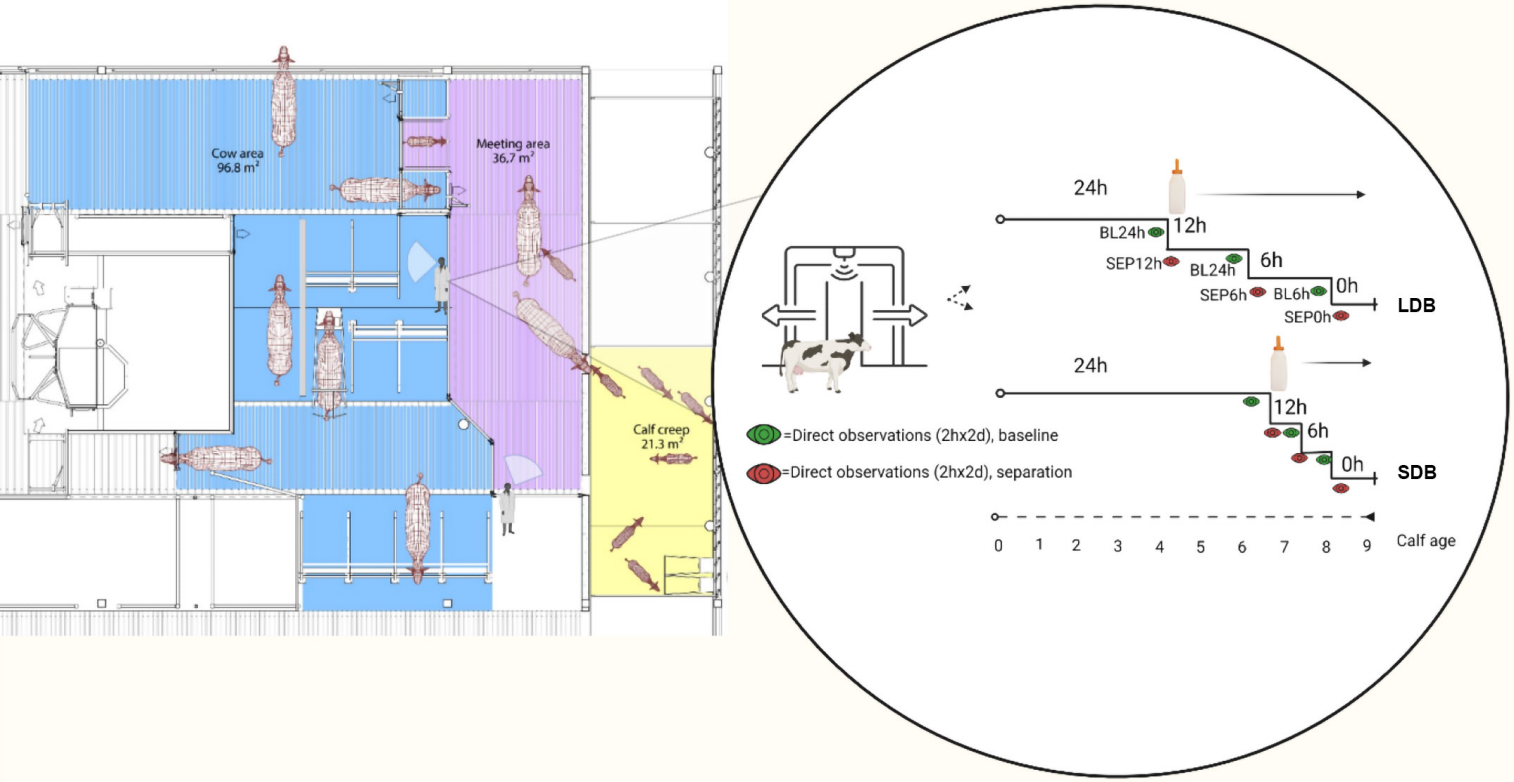
This timeline shows when behavioral observations were carried out in a trial studying 2 debonding strategies using 30 cow-calf pairs in which cows initially had 24 h/d smart-gate access to a meeting area (and their calves). For short debonding (SDB) pairs, a reduction in cow access commenced at calf age of 6.5 wk through 5 d of 12 h/d access followed by 5 d of 6 h/d access. For long debonding (LDB) pairs, a reduction in cow access commenced at calf age of 4 wk through 14 d of 12 h/d access followed by 14 d of 6 h/d access. For both treatments, supplemental milk was provided once cow access was reduced and cows were prevented any access in wk 8–9. BL = baseline; SEP = separation.

Calves could suckle freely once cows entered the meeting area. Individualized access to supplemental whole milk (12 L/d, CF500S, DeLaval International AB, Tumba, Sweden) was granted once cow smart-gate access was reduced to 12 h/d. All calves were weaned off milk during the last 4 d of the study. Calves always had access to water and roughage (hay, and during the last week also grass silage) and concentrate from the automatic feeder.

The data used to study our aims were collected at the individual cow and calf level. Behaviors were recorded during direct observations for 120 min on each of 2 consecutive days (= phases) before (baseline: **BL24h**, **BL12h**, and **BL6h**) and after smart-gate settings were changed to accommodate reduced cow access (separation: **SEP12h**, **SEP6h**, **SEP0h**). We recorded high- and low-pitched vocalizations continuously (Johnsen et al., 2015b). Using scan sampling/minute, we recorded if a focal cow or calf was positioned close (<1 m) to the separation barrier during any of the 120 observed minutes. By design, the observers were not blind to the treatments.

Daily records of individual calf supplemental milk intake were obtained from the automatic feeders using DelPro software (DeLaval International AB). In the current study, we only used individual milk intakes dichotomized for low versus higher intakes of milk (≤1.5 L/d vs. >1.5 L/d; Johnsen et al., 2016), recorded in the different phases as explained above (SEP12h, BL12h, SEP6h, BL6h, and SEP0h).

The outcomes of interest were high- and low-pitched vocalizations and positions of the cow and calf, respectively. We summed number of vocalizations and registrations of position <1 m per observation day and used averages across the 2 consecutive BL or SEP days in a phase, individuals and batches in a treatment. Separate mixed linear regression models were built using Stata SE/16 (Stata Corp., College Station, TX) with animal ID and batch included as random effects. Fixed effects were treatment (LDB and SDB) and phase (BL24h, SEP12h, BL12h, SEP6h, BL6h, and SEP0h) and their interaction. Post hoc, we used the *margins* command to contrast the treatments within each phase (Bartus, 2005). In a subset of data (excluding BL24h since no supplemental milk was available in this phase), the impact of supplemental milk on calf high-pitched vocalizations was assessed in a separate model with milk intake (low vs. high), phase, and their interaction as fixed effects. Throughout, we specified the within-subject covariance structure as autoregressive and applied the function *vce robust* to account for repeated measures. Significance was declared at *P* < 0.05 and tendencies at *P* < 0.10.

Upon restrictions of gate access, calves and cows responded with performing high-pitched vocalizations (phase *P* < 0.01 for both calves and cows, Figure 2). The same increase with phase was seen for calf (but not cow) low-pitched vocalizations and for time spent close to the separation barrier (Table 1). We saw great individual variation in the behavioral responses (see graphical abstract). Overall, no main effect of treatment indicates only subtle differences for the studied cow behaviors and for high-pitched calf vocalizations. However, LDB calves spent less time close to the separation barrier and emitted more low-pitched vocalizations. Both calves' and cows' high-pitched vocal response varied differently with treatment and phase (treatment × phase *P* < 0.01 for calves and *P* = 0.036 for cows). Compared with that of SDB, LDB calves (but not cows) produced fewer high-pitched vocalizations initially, at SEP12h (*P* = 0.042 and *P* = 0.814 for calves and cows, respectively). Once cows no longer had any access to the calves (SEP0h), LDB calves spent more time close to the separation barrier than SDB calves. Similarly, LDB cows (but not calves) produced more high- and low-pitched vocalizations during BL6h, whereas a tendency for the same pattern was found in SEP0h. Calves that consumed >1.5 L supplemental milk/d performed less high-pitched vocalizations in all phases except SEP6h.

**Figure 2:**
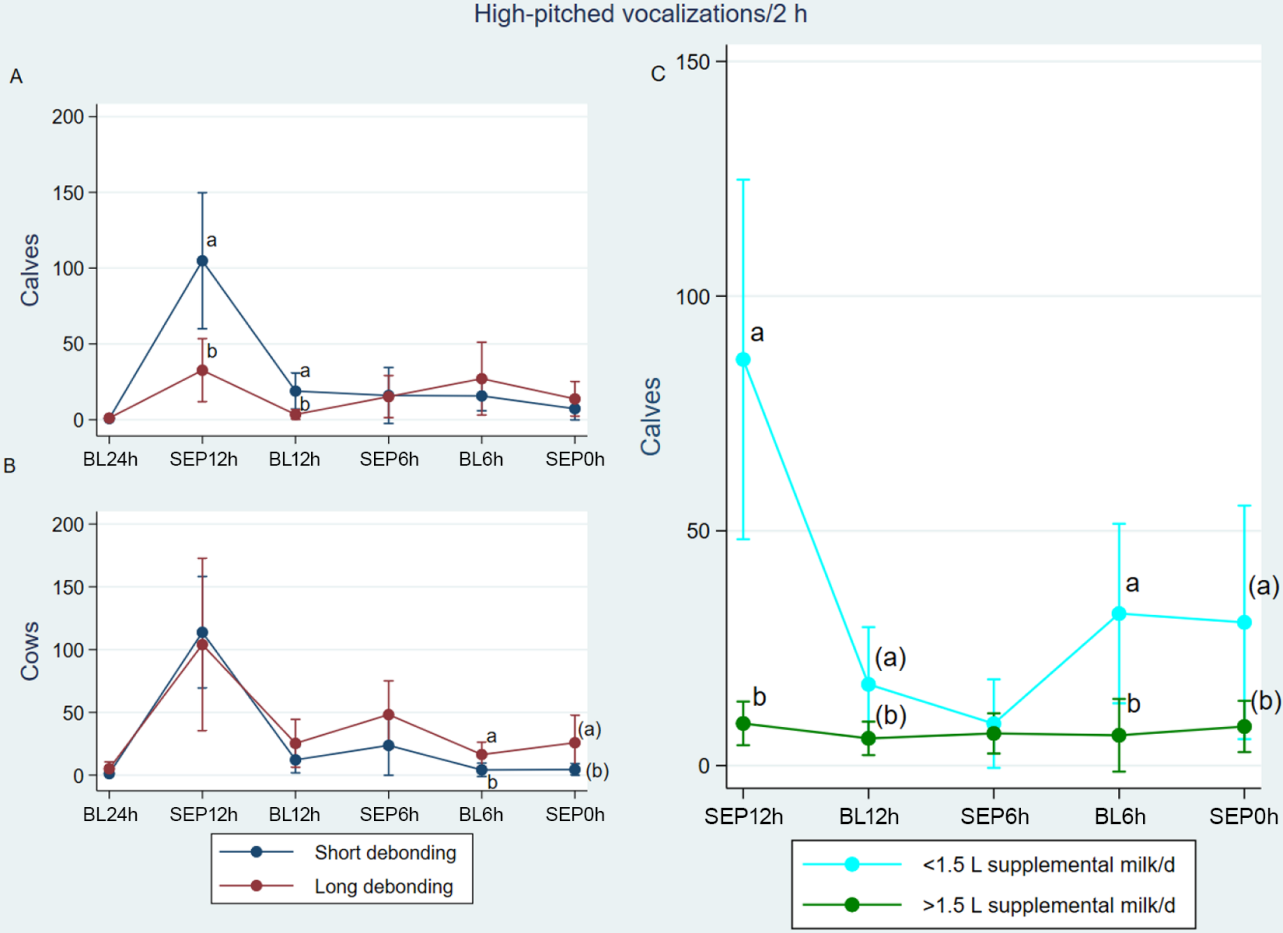
These margin plots show the predicted high-pitched vocal response of (A) calves and (B) cows in response to a gradual decrease in the cows' access to the meeting area in a cow-directed cow-calf contact system. Error bars represent the SE. We compared 2 debonding methods using 30 cow-calf pairs. Cows initially had 24 h/d smart-gate access to a meeting area (and their calves). Direct observations (2 h × 2 d) were performed during 24 h/d access (baseline, BL24h), and thereafter once cow access was reduced (separation, SEP). For short debonding pairs, a reduction in cow access commenced after 6.5 wk through 5 d of 12 h/d access followed by 5 d of 6 h/d access. For long debonding pairs, a reduction in cow access commenced after 4 wk through 14 d of 12 h/d access followed by 14 d of 6 h/d access. For both treatments, cows were prevented any access in wk 8–9, SEP0h). In (C), calf high-pitched vocal response is shown separately for calves depending on their individual daily intake of supplemental milk from the milk feeder (> or <1.5 L milk/d). Differing letters indicate significant differences (*P* ≤ 0.05) or tendencies (in parentheses, *P* ≤ 0.10).

**Table 1:** Effects of long (initially 4 wk of free access to a meeting area followed by 14 d of 12 h access + 14 d of 6 h access) versus short (initially 6.5 wk of free access followed by 5 d of 12 h access + 5 d of 6 h access) debonding on cow and calf reinstatement behavior registered during direct observations and presented as least squares means (SE) across the observations within phase (2 h × 2 d)

Behavior	Phase^2^	Treatment^1^												*P*-value		
		Long debonding						Short debonding								
		BL24h	SEP12h	BL12h	SEP6h	BL6h	SEP0h	BL24h	SEP12h	BL12h	SEP6h	BL6h	SEP0h	Trt^1^	Phase	Trt × phase
Low-pitched vocalizations/2 h	Cow	5.1	104.0	25.3	48.2	16.5^a^	25.8^(a)^	1.2	113.8	12.2	23.7	4.2^b^	4.5^(b)^	0.174	<0.1738	0.036
		(2.8)	(35.0)	(9.7)	(13.7)	(5.0)	(11.1)	(0.7)	(2.6)	(5.3)	(12.2)	(2.7)	(2.3)			
	Calf	1.4^a^	9.3	3.1	11.6^(a)^	10.6	10.1	0.2^b^	7.63	5.5	2.5^(b)^	4.5	1.4	0.0252	<0.001	0.015
		(0.5)	(3.7)	(1.1)	(4.1)	(4.3)	(7.0)	(0.1)	(2.0)	(1.4)	(1.1)	(1.8)	(0.5)			
Time spent close, %	Cow	2.2	11.7	5.9	9.4	5.0	3.6	3.1	13.0	7.8	5.5	2.78	1.72	0.415	<0.001	0.105
		(0.8)	(2.6)	(1.8)	(2.2)	(1.7)	(2.7)	(0.7)	(2.2)	(1.8)	(1.7)	(1.1)	(0.5)			
	Calf	11.9^a^	12.1^a^	15.7^a^	27.1	22.0^a^	17.9^a^	5.6^b^	19.4^b^	28.6^b^	20.8	38.5^b^	8.0^b^	0.001	<0.001	<0.001
		(1.6)	(1.2)	(1.6)	(2.9)	(4.0)	(3.4)	(1.0)	(2.6)	(3.4)	(3.0)	(4.9)	(1.5)			

a,b Mean values within the same row and phase with different superscripts differ (*P* < 0.05) for the interaction between treatment and phase. Tendencies (*P* < 0.1 ≥ 0.05) are indicated with parentheses [e.g., (a)].

1 Treatment (Trt) = long debonding or short debonding). During the last week of the trial, cows had no access to the meeting area (SEP0h).

2 Phase = phases are indicated by baseline (BL) dependent on whether or not the direct observation carried out denotes a time point in which the cows and calves are expected to be habituated to the gradual reductions in cow access from free (BL24h) to, for example, SEP12h (first 2 d after cow access to the meeting area is reduced from 24 to 12 h).

Cow-calf contact systems have the potential to allow for a behavioral repertoire implicit in a maternal-filial relationship (Sirovnik et al., 2020; EFSA Panel on Animal Health and Animal Welfare et al., 2023) and evoke positive subjective states through a complex bond (Boissy et al., 2007). However, separation stress should not outweigh these benefits. As expected, cow-calf pairs responded to a decreased cow access to the meeting area with emitting high- and low-pitched vocalizations, and spending more time close to the separation barrier. These behaviors peaked at the first instance of decreasing contact (SEP12h). This likely indicates (unfulfilled) anticipation of cow-calf reunion in a cow-driven CCC system as described for other CCC systems (e.g., Roadknight et al., 2022; Wenker et al., 2022). We have previously shown that cows' high-pitched vocal (stress) response is positively associated with the number of unrewarded smart-gate visits (Johnsen et al., 2021).

For the main indicator of separation stress, high-pitched vocalizations, individual variation among calves and cows was higher than the between-treatment effects. This finding supports Wenker et al. (2022) in suggesting individualized separation protocols. The initial lowered acute high-pitched vocal response of long debonding calves in this study likely reflects an age effect rather that a treatment effect since treatments did not differ vastly at this point (first decrease of cow access). Separation of cow and calf is stressful regardless of when it occurs (Weary et al., 2008), but these results indicate that 4-wk-old (LDB) calves show less behavioral indicators of stress than 6.5-wk-old (SDB) calves, perhaps echoing previous findings (Weary and Chua, 2000; Stěhulová et al., 2008). However, this pattern of a milder behavioral response was sustained since LDB calves still vocalized less at BL12h, possibly indicating that calves may benefit from a longer debonding period. The finding that LDB calves spent less time close to the separation barrier may also indicate a “milder” response for calves debonded earlier and at a slower pace. This may be linked to younger calves being more flexible to learn to drink from artificial teats and thus eased development of nutritional independence (Johnsen et al., 2015a) or more readily habituating to the new “visiting pattern” of the cows. For the cows, a longer debonding period may have evoked a second (lower) peak in separation stress response since LDB cows emitted more high-pitched vocalizations than SDB cows once all conditions were similar (both treatments had 0 h contact).

Both cows and calves continued to spend time close to the separation barrier and emit low-pitched vocalizations in the last week of the study which may indicate that the social bond between dam and calf is sustained (Veissier et al., 1990). The increased low-pitched vocalizations of LDB calves may also indicate a more positively valenced contact toward the dam (Green et al., 2021; Schnaider et al., 2022), perhaps because the time between new reductions in cow access appeared further away in time (every 14 d) than for SDB calves (every 5 d). Future studies may disentangle the calf age effect from the length of debonding.

From the cows' perspective, this study did not find clear advantages for one debonding method over the other. A clear behavioral response to separation from the calf is expected given the parent-offspring conflict theory and the nutritional independence associated with the premature separation (9 wk in this study) versus natural weaning (8–12 mo, Reinhardt and Reinhardt, 1981). Even though LDB cows were given ample time getting used to reduced access, a response (i.e., high-pitched vocalizations) was still evoked when access was further reduced to 6 h and 0 h. A nuanced discussion of cow separation distress in CCC systems is needed to disentangle and balance the needs of all involved stakeholders (cow, calf, dairy farmer, and so on).

It was also expected that calves consuming supplemental milk during separation emitted fewer high-pitched vocalizations (Johnsen et al., 2015a). Thus, vocal response increases with age at separation, but is modulated by nutritional independence. Although teaching calves to drink milk from a new milk source (e.g., automatic milk feeder) is connected with some workload, this is associated with less calf stress. Together with other evidence of individuality in feed intake preweaning (e.g., Neave et al., 2019; Whalin et al., 2022), these results support an individualized separation protocol for calves, which is possible in computer-controlled cow or calf-directed CCC systems.

In conclusion, a longer debonding period initiated at a lower age before separation may alleviate the initial behavioral response to separation, especially for calves. The vocal response of calves increases with age at separation but is modulated by intake of supplemental milk.
